# Far-field multi-beam pattern synthesis for phased array antennas using Lorentz reciprocity theorem

**DOI:** 10.1371/journal.pone.0343372

**Published:** 2026-02-24

**Authors:** Yida Fan, Lijuan Li, Ravi Kumar Arya, Junwei Dong, Shiyuan Kong

**Affiliations:** 1 The School of Optoelectronic Engineering, Changchun University of Science and Technology, Changchun, Jilin, China; 2 Xiangshan Laboratory, Zhongshan Institute of Changchun University of Science and Technology, Zhongshan, Guangdong, China; Guangdong University of Petrochemical Technology, CHINA

## Abstract

In this paper, by combining the Lorentz reciprocity theorem with the particle swarm algorithm, the array beam synthesis method based on wireless power transmission efficiency is successfully applied to the automated beam synthesis process. The improved particle swarm algorithm can achieve automatic gain balancing among multiple beams. This method explicitly combines the radiation pattern of the array element to construct the scattering parameter matrix, providing a new approach in addition to simulation software calculation and experimental measurement, and improving the conventional method of maximum power transmission efficiency. For experimental validation, a 2.45 GHz phased array antenna with a dual-layer feed structure is designed and fabricated. The proposed algorithm successfully synthesizes 2D spatially scanned beams, as well as balanced-gain dual-beam and quad-beam patterns, with measured gain variations below 0.1 dB and computational optimization completed within 8 seconds. Experimental results demonstrate precise beam steering at target angles while maintaining exceptional gain uniformity across all beams. The solution’s pattern-dependent framework ensures universal applicability to diverse array configurations requiring multi-beam synthesis with scanning capability and gain equalization, offering a robust tool for next-generation communication and radar systems.

## Introduction

With the rapid development of wireless communication systems, radar detection, and satellite navigation technologies, phased array antennas have emerged as one of the core components in modern wireless systems due to their beam controllability, high gain, and spatial resolution capabilities [1]. Multi-beam phased array antennas enable simultaneous generation of multiple independently controllable beams with high-speed beam steering to cover distinct spatial regions or serve multiple users. Correspondingly, multi-beam pattern synthesis methodologies have gained extensive applications in radar sensors, radio astronomy, and satellite communications, demonstrating significant theoretical and practical value [2].

Pattern synthesis research primarily follows two approaches: hardware-circuit implementation through beamforming network design combined with matrix switching to achieve radiation pattern control, though this method introduces additional spatial constraints and higher costs by requiring extra beamforming circuit layers within the antenna structure [3–8]; and algorithmic optimization techniques that derive optimized array excitation coefficients through computational methods for direct beam synthesis via integrated phase shifters and attenuators, offering enhanced flexibility and cost-effectiveness [9–14].

Traditional analytical methods such as Chebyshev and Taylor synthesis approaches provide rapid and effective solutions but exhibit substantial application limitations in modern scenarios [15–17]. While these classical algorithms remain foundational, their integration with optimization techniques has become imperative to address evolving application requirements. Numerical optimization algorithms, including genetic algorithms, particle swarm optimization (PSO), and simulated annealing, demonstrate broad applicability in multi-beam challenges [18–21], though their efficacy critically depends on internal workflow design, where poorly structured implementations risk convergence to local optima and optimization failure. Convex optimization techniques excel at precisely satisfying multi-constraint conditions involving main lobe width and sidelobe level suppression while achieving global optima within constrained timeframes [22–24], yet their performance remains sensitive to initial parameter selection, necessitating empirical knowledge or hybrid initialization strategies to prevent convergence failures. Recent advancements in neural networks and deep learning-based pattern synthesis methods have enabled dynamic beam adaptation through data-driven learning of excitation-pattern mappings, though their practical implementation faces inherent dependencies on the quality and quantity of training data – insufficient datasets may induce beam distortion and algorithmic instability [25–28].

The method of Maximum Power Transmission Efficiency (MMPTE) has emerged as an innovative antenna design approach that optimizes wireless system power transmission efficiency through direct analytical solutions of array excitations maximizing multi-antenna system efficiency models [29–31], bypassing iterative optimization processes and introducing a novel paradigm for pattern synthesis [32,33]. However, a fundamental constraint arises from the algorithm’s prerequisite for precise multi-port network scattering parameter (S-parameter) matrix inputs, which typically necessitates acquisition through computationally intensive full-wave electromagnetic simulations or experimental characterization, thereby substantially restricting its practical implementation in pattern synthesis applications requiring rapid design iterations or dynamic reconfiguration capabilities.

This paper proposes an enhanced multi-beam synthesis methodology for array antennas that significantly improves the performance of MMPTE through a high-efficiency, low-complexity approach. By employing the Lorentz reciprocity theorem to determine transmission coefficients between transmit-receive antenna pairs, we computationally construct multi-port network scattering parameter matrices, effectively reducing dependence on full-wave simulations or experimental measurements. The methodology integrates an advanced particle swarm optimization algorithm with MMPTE to extract optimal weighting matrices that achieve beam gain equalization. Our comprehensive framework synergistically combines the Lorentz reciprocity theorem, MMPTE, and intelligent optimization to establish a novel synthesis paradigm where radiation patterns of array elements and beam positioning requirements serve as inputs, generating optimized array excitation coefficients as outputs. This integrated solution provides an efficient excitation calculation mechanism for cost-effective phased array systems requiring multi-beam generation and beam scanning capabilities while maintaining system simplicity and computational tractability.

The remainder of this paper is organized as follows: Section II details the algorithmic implementation framework, encompassing the derivation of scattering parameters via the Lorentz reciprocity theorem, the multi-beam synthesis methodology optimizing power transfer efficiency, and the gain equalization strategy employing enhanced particle swarm optimization for weighting factor determination. Section III elaborates the phased array antenna design methodology, including array architecture development, RF front-end component integration, and comprehensive link calibration procedures. Section IV evaluates the proposed algorithm’s performance through a systematic analysis of the implemented phased array system. Finally, Section V concludes the paper with a concise summary of key technical contributions and implementation outcomes, followed by discussions on potential applications and future research directions.

## Multi-beam synthesis method using antenna spherical far-field pattern

This section is structured into three subsections, each addressing a distinct methodology: 1) solving scattering parameters based on the reciprocity theorem, 2) multi-beam synthesis based on power transmission efficiency, and 3) a dynamic PSO-based beam gain enhancement method. Collectively, these subsections comprehensively elaborate the implementation framework for array antenna multi-beam synthesis.

### Solving scattering parameters based on reciprocity theorem

**Fig 1:**
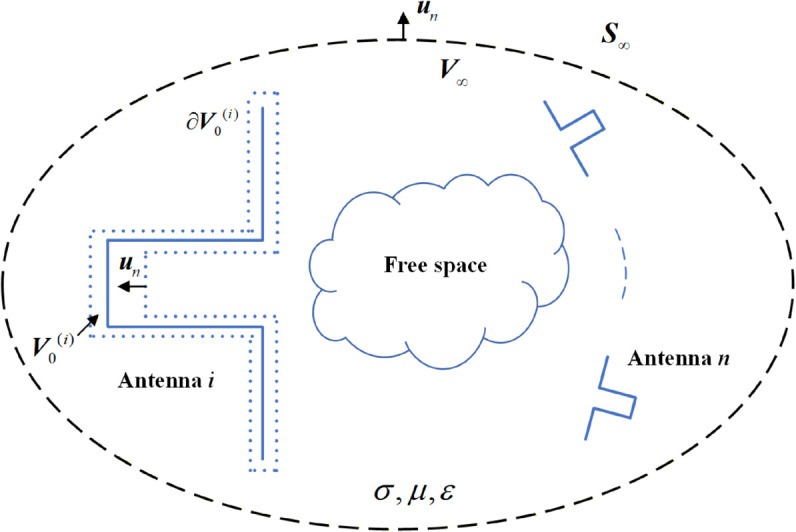
Multi-antenna scattering scenario.

Consider a system consisting of *n* antennas contained in a region $V_{\infty }$ bounded by $S_{\infty }$, where $S_{\infty }$ does not include any field source surfaces. The scenario described is shown in Fig 1.

Let *Z*_*sl*_ be the reference impedance for the input terminal of antenna *l*, the voltage and current of the antenna *l* can be represented by normalizing the input of the incident wave and the reflected wave as:

Vl(i)=Z¯slReZslal(i)+ZslReZslbl(i),Il(i)=1ReZslal(i)−1ReZslbl(i), (1)

where $V_{l}^{\left(i\right)}$ and $I_{l}^{\left(i\right)}$ represents the voltage and current on the surface of antenna *l* when antenna *i* is transmitting and other antennas are receiving.

Under ideal conditions, for a single-mode fed waveguide, we have:

$$\int_{\partial V_{0}^{\left(i\right)}}\left({𝐄}_{i}\times {\bar{𝐇}}_{i}\right)\cdot {𝐮}_{n}dS=-V_{l}^{\left(i\right)}{\bar{I}}_{l}^{\left(i\right)},l=1,2,\cdots ,n.$$ (2)

where ${𝐄}_{i}$ and ${𝐇}_{i}$ are defined as follows, ${𝐞}_{1l}(x,y)$ is a vector function of the dominant mode in the feed waveguide of the antenna *l*.

$${𝐄}_{i}(𝐫)=V_{l}^{\left(i\right)}{𝐞}_{1l}(x,y),{𝐇}_{i}(𝐫)=I_{l}^{\left(i\right)}{𝐮}_{z}\times {𝐞}_{1l}(x,y)$$ (3)

According to frequency-domain reciprocity [34,35]:

$$\int_{S}\left({𝐄}_{i}\times {𝐇}_{j}-{𝐄}_{j}\times {𝐇}_{i}\right)\cdot {𝐮}_{n}dS=0$$ (4)

We can get:

$$\;\sum_{l=1}^{n}\int_{\partial V_{0}^{\left(i\right)}}\left({𝐄}_{i}\times {𝐇}_{j}-{𝐄}_{j}\times {𝐇}_{i}\right)\cdot {𝐮}_{n}dS\;+\int_{S_{\infty }}\left({𝐄}_{i}\times {𝐇}_{j}-{𝐄}_{j}\times {𝐇}_{i}\right)\cdot {𝐮}_{n}dS\;=\sum_{l=1}^{n}\left[V_{l}^{\left(j\right)}I_{l}^{\left(i\right)}-V_{l}^{\left(i\right)}I_{l}^{\left(j\right)}\right]\;=\sum_{l=1}^{n}\left[a_{l}^{\left(i\right)}b_{l}^{\left(j\right)}-a_{l}^{\left(j\right)}b_{l}^{\left(i\right)}\right]=0,$$ (5)

where $S=S_{\infty }+\sum_{l=1}^{n}\partial V_{0}^{\left(l\right)}$.

In order to represent the scattering parameter between two antennas, we use $S=S_{i}^{\prime }$  +  $\partial V_{0}^{\left(i\right)}$, where *S* = *S*_*i*_ is a closed surface containing antenna *i* only. According to Eq (4), we have:

$$\;\int_{\partial V_{0}^{\left(i\right)}}\left({𝐄}_{i}\times {𝐇}_{j}-{𝐄}_{j}\times {𝐇}_{i}\right)\cdot {𝐮}_{n}dS\;+\int_{S_{i}^{\prime }}\left({𝐄}_{i}\times {𝐇}_{j}-{𝐄}_{j}\times {𝐇}_{i}\right)\cdot {𝐮}_{n}dS=0,$$ (6)

Further combining Eqs (3) and (6), it yields:

$$V_{i}^{\left(i\right)}I_{i}^{\left(j\right)}-V_{i}^{\left(j\right)}I_{i}^{\left(i\right)}=\int_{S_{i}^{\prime }}\left({𝐄}_{i}\times {𝐇}_{j}-{𝐄}_{j}\times {𝐇}_{i}\right)\cdot {𝐮}_{n}dS$$ (7)

If all other antennas l(l≠i) are in an open-circuit state while antenna *i* is transmitting, that is, $I_{l}^{\left(i\right)}=0$ for l≠i,

$$V_{i}^{\left(j\right)}I_{i}^{\left(i\right)}=-\int_{S_{i}^{\prime }}\left({𝐄}_{i}\times {𝐇}_{j}-{𝐄}_{j}\times {𝐇}_{i}\right)\cdot {𝐮}_{n}dS=V_{j}^{\left(i\right)}I_{j}^{\left(j\right)},\;b_{i}^{\left(j\right)}a_{i}^{\left(i\right)}=-\frac{1}{2}\int_{S_{i}^{\prime }}\left({𝐄}_{i}\times {𝐇}_{j}-{𝐄}_{j}\times {𝐇}_{i}\right)\cdot {𝐮}_{n}dS=b_{j}^{\left(i\right)}a_{j}^{\left(j\right)},$$ (8)

Similarly,

$$V_{j}^{\left(i\right)}I_{j}^{\left(j\right)}=-\int_{S_{j}^{\prime }}\left({𝐄}_{j}\times {𝐇}_{i}-{𝐄}_{i}\times {𝐇}_{j}\right)\cdot {𝐮}_{n}dS=V_{i}^{\left(j\right)}I_{i}^{\left(i\right)},\;b_{j}^{\left(i\right)}a_{j}^{\left(j\right)}=-\frac{1}{2}\int_{S_{j}^{\prime }}\left({𝐄}_{j}\times {𝐇}_{i}-{𝐄}_{i}\times {𝐇}_{j}\right)\cdot {𝐮}_{n}dS=b_{i}^{\left(j\right)}a_{i}^{\left(i\right)},$$ (9)

Further combining Eqs (8) and (9), finally we can get:

$$S_{ij}={\frac{b_{i}^{\left(j\right)}}{a_{j}^{\left(j\right)}}|}_{a_{l}^{\left(j\right)}=0,l\neq j}\;=-\frac{1}{2a_{i}^{\left(i\right)}a_{j}^{\left(j\right)}}\int_{S_{i}^{\prime }}\left({𝐄}_{i}\times {𝐇}_{j}-{𝐄}_{j}\times {𝐇}_{i}\right)\cdot {𝐮}_{n}dS\;=S_{ji}.$$ (10)

**Fig 2:**
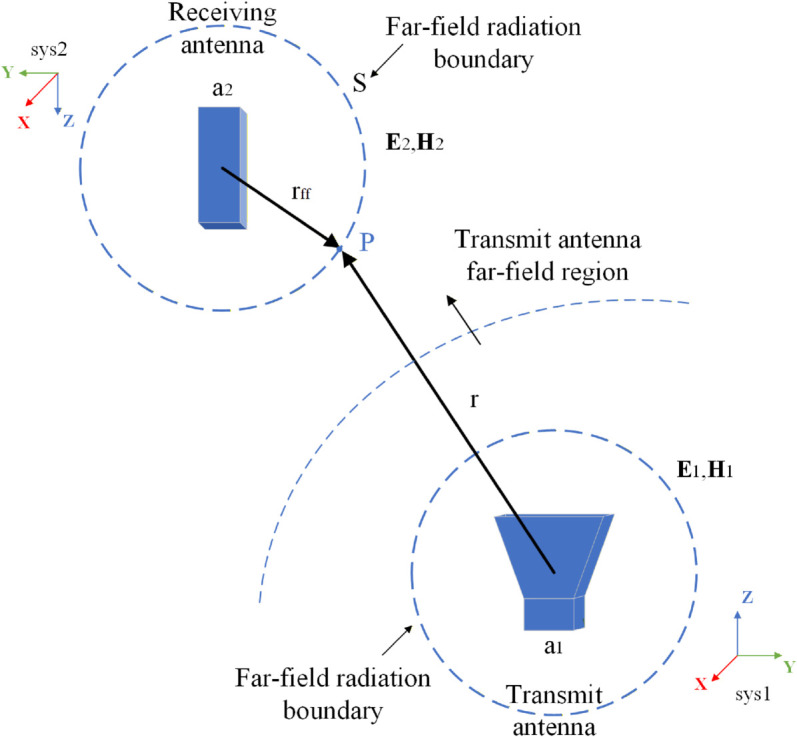
A scattering parameter solving model.

In order to calculate the scattering parameters of two antennas, we need to know the independent radiation patterns of the two antennas. The receiving antenna is placed in the far field region of the transmitting antenna, and a scattering parameter solving model between the two antennas can be shown in Fig 2. *E* and *H* are respectively taken from the far field radiation pattern of the two antennas. According to the calculation equation of the far field electric field, *E* and *H* are expressed as Eq (11):

$$𝐄(𝐫)=\frac{e^{-jkr}}{r}\left[{𝐄}_{\infty }\left({𝐮}_{r}\right)+O\left(\frac{1}{r}\right)\right],\;𝐇(𝐫)=\frac{e^{-jkr}}{r}\left[{𝐇}_{\infty }\left({𝐮}_{r}\right)+O\left(\frac{1}{r}\right)\right],$$ (11)

According to the propagation characteristics of electromagnetic waves in free space, electric and magnetic fields have the following relations:

$$\sqrt{\frac{\mu }{\epsilon }}{𝐇}_{\infty }\left({𝐮}_{r}\right)={𝐮}_{r}\times {𝐄}_{\infty }\left({𝐮}_{r}\right)$$ (12)

For the application of Eq (10), it is necessary to map the field of the transmitting antenna to the receiving antenna coordinate system (sys2) based on the rotation matrix of the coordinate system and the transformation matrix of the rectangular coordinate system to the spherical coordinate system. The *S* in Fig 2 represents the surface of a sphere region capable of enclosing the entire receiving antenna structure, *r*_*ff*_ is the radius of *S*.

Mapping the electromagnetic field vector from the transmitting antenna coordinate system (sys1) to the receiving antenna coordinate system (sys2) follows the rotation matrix formula,


$$\left[\begin{array}{c} {\hat{x}}^{{\;\;}^{\prime }}\\ {\hat{y}}^{{\;\;}^{\prime }}\\ {\hat{z}}^{{\;\;}^{\prime }} \end{array}\right]=R_{z}(\gamma )\cdot R_{y}(\beta )\cdot R_{x}(\alpha )\left[\begin{array}{c} \hat{x}\\ \hat{y}\\ \hat{z} \end{array}\right]$$

$$R_{x}(\alpha )=\left[\begin{array}{ccc} 1 & 0 & 0\\ 0 & \cos\alpha & -\sin\alpha \\ 0 & \sin\alpha & \cos\alpha \end{array}\right]$$

$$R_{y}(\beta )=\left[\begin{array}{ccc} \cos\beta & 0 & \sin\beta \\ 0 & 1 & 0\\ -\sin\beta & 0 & \cos\beta \end{array}\right]$$

$$R_{z}(\gamma )=\left[\begin{array}{ccc} \cos\gamma & -\sin\gamma & 0\\ \sin\gamma & \cos\gamma & 0\\ 0 & 0 & 1 \end{array}\right]$$ (13)

The electric and magnetic fields are denoted respectively by the $\left(\hat{r},\hat{\theta },\hat{\phi }\right)$ components as $\left(E_{r},E_{\theta },E_{\phi }\right)$, and Eq (10) can be rearranged as:

$$S_{21}=-\frac{1}{2a_{1}a_{2}}\int_{S}\left(E_{2}\times H_{1}-E_{1}\times H_{2}\right)\cdot \hat{r}r^{2}\sin\theta d\theta d\varphi \;=-\frac{1}{2\sqrt{Z_{s1}}\sqrt{Z_{s2}}}\int_{\theta =0}^{\pi }\int_{\varphi =0}^{2\pi }[\left(E_{\theta 2}\cdot H_{\varphi 1}-E_{\varphi 2}\cdot H_{\theta 1}\right)\;\;-\left(E_{\theta 1}\cdot H_{\varphi 2}-E_{\varphi 1}\cdot H_{\theta 2}\right)]r^{2}\sin\theta d\theta d\varphi$$ (14)

In constructing the electric field model for the radiating element of the transmitting antenna, the influence from other adjacent array elements in its vicinity has been taken into account. Typically, structures located closer to the antenna from which the electric field is extracted have a more significant impact on its radiation characteristics. When extracting the electric field distribution data of the transmitting element, a pre-established 3 × 3 array model—arranged with the same periodicity as the antenna array—was constructed around the element. The element under study is positioned at the center of this array, thereby ensuring that the extracted electric field data approximately incorporates interference factors arising from mutual radiative interactions among the elements.

### Multi-beam synthesis based on power transmission efficiency

**Fig 3:**
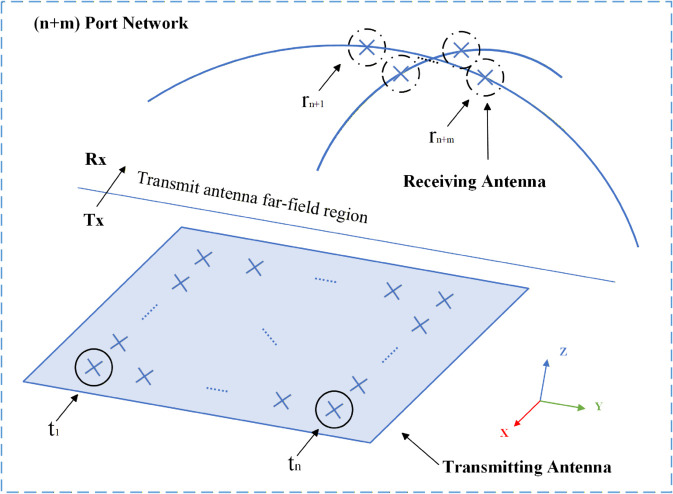
A wireless power transmission system.

Let’s consider an array antenna and a set of virtual receiving antennas as an array antenna power transmission system in a natural environment, as shown in Fig 3. Concentrating the radiation pattern of the array antenna within a specific region is equivalent to focusing the transmitted power from the antenna array onto a designated virtual receiving antenna. Within the array antenna-based power transmission system, the combination of *n* transmitting ports and *m* virtual receiving ports forms a unified *n* + *m*-port network [36].

The multi-beam synthesis problem can be equivalently formulated as optimizing the efficiency of power transmission from an array antenna to virtual receiving antennas at multiple spatial directions. Based on the angular positions of these virtual receivers, beamforming can be synthesized to precisely steer radiation toward their respective orientations.

The matrices [*A*] and [*B*] are constructed, which can be represented by the following equation:

$$\left[A]={\left[S_{rt}\right]}^{H}{\left[W\right]}^{H}[W][S_{rt}]\;[B]=1-{\left[S_{tt}\right]}^{H}[S_{tt}\right]$$ (15)

where [*S*_*tt*_] is the scattering parameter matrix among all transmitting array elements, the dimension of the matrix is N×N, which contains the coupling coefficients between the array ports. [*S*_*rt*_] is the scattering parameter matrix between all virtual receiving antennas and all transmitting array antennas, and the dimension of the matrix is M×N. [*W*] is the weight of the gain distribution among all virtual receiving antennas, and is a diagonal matrix with the same dimension as the number of virtual receiving antennas; the dimension of [*W*] is M×M. The excitation of the array antenna is expressed as [*a*_*t*_] and the dimension is N×1.

The power transmission efficiency (PTE) function of the array antenna power transmission system is expressed as:

$$\eta =\frac{{\left[a_{t}\right]}^{H}[A][a_{t}]}{{\left[a_{t}\right]}^{H}[B][a_{t}]}$$ (16)

‌‌By leveraging the Rayleigh quotient representation of the reception efficiency *η* for virtual receiving antennas in the system, we can determine the optimal excitation coefficients of the array antenna system that maximize the reception efficiency, thereby achieving multi-beam synthesis. The excitation inputs of the array antenna can be expressed as:

$${\left[a_{t}\right]}^{*}=\arg\max_{[a_{t}]\neq 0}\frac{{\left[a_{t}\right]}^{H}[A][a_{t}]}{{\left[a_{t}\right]}^{H}[B][a_{t}]},\;s.t.{\left[B\right]}^{-1}[A]{\left[a_{t}\right]}^{*}=\lambda_{\max}{\left[a_{t}\right]}^{*}\;$$ (17)

### Dynamic particle swarm optimization-based beam gain enhancement method

The PSO algorithm can rapidly converge to particle positions that satisfy the objective function [37]. By leveraging Eqs (14) and (17), the PSO framework can be integrated with the radiation pattern distribution function to iteratively search for the optimal weighting matrix [*W*], thereby deriving the required array excitation coefficients.

The gain distribution among multiple beams can be dynamically regulated by adjusting the weighting matrix $\left[W]=diag(w_{1},w_{2},\ldots ,w_{m}\right)$, therefore, the particle of the PSO algorithm is defined as $\left[w_{1},w_{2},\ldots ,w_{m}\right]$. The PSO optimization method follows the following equation:

$$v_{i}=\omega \times v_{i}+c_{1}\times \mathrm{rand}()\times \left({\text{pbest}}_{i}-x_{i}\right)\;\;\;\;+c_{2}\times \mathrm{rand}()\times \left({\text{gbest}}_{i}-x_{i}\right)\;x_{i}=x_{i}+v_{i}$$ (18)

The inertia factor *ω* and learning factors $c_{1},c_{2}$ decrease linearly with the increase of iteration rounds, so that the algorithm can achieve both global and local optimization.

$$w(t)=w_{\max}-\frac{\left(w_{\max}-w_{\min}\right)\cdot t}{T_{\max}}\;c_{1}(t)=c_{1,\max}-\frac{\left(c_{1,\max}-c_{1,\min}\right)\cdot t}{T_{\max}}\;c_{2}(t)=c_{2,\min}+\frac{\left(c_{2,\max}-c_{2,\min}\right)\cdot t}{T_{\max}}$$ (19)

To mitigate premature convergence in particle swarm optimization, the algorithm dynamically monitors population diversity by comparing real-time calculated diversity metrics against a predefined local optima threshold throughout the search process. When the diversity index falls below the critical threshold, a recovery mechanism is triggered to randomly reset positions of particles trapped in local optima, with the quantity of reset particles governed by a configurable reset rate parameter. This adaptive diversity preservation strategy effectively alleviates stagnation in suboptimal solutions while enhancing probabilistic access to global optimum solutions. The mathematical formulation of population diversity is defined as follows:

$$\text{ Diversity }=\frac{1}{N}\sum_{i=1}^{N}\left\|{𝐱}_{i}-\overset{―}{𝐱}\right\|$$ (20)

Generally, to maintain a predefined inter-beam gain allocation ratio, the gains between beams must satisfy the following relationship:

$$\frac{G\left(\theta_{1},\phi_{1}\right)}{\alpha_{1}}=\frac{G\left(\theta_{2},\phi_{2}\right)}{\alpha_{2}}=\cdots =\frac{G\left(\theta_{m},\phi_{m}\right)}{\alpha_{m}}$$ (21)

G(θ,ϕ) represents the value of the array antenna radiation pattern at (θ,ϕ), *α* represents the relative gain ratio of multiple beams. For simple ideal conditions, the radiation pattern can be simply represented using array factors:

$$G(\theta ,\varphi )={\left[a_{t}\right]}^{H}[a(\theta ,\phi )]{\left[a(\theta ,\phi )\right]}^{H}[a_{t}]$$ (22)

where [a(θ,ϕ)] is a N×1 dimensional matrix,

$$a(\theta ,\varphi )=\left[\begin{array}{c} e^{jk(x_{1}\sin\theta \cos\varphi +y_{1}\sin\theta \sin\varphi +z_{1}\cos\theta )}\\ \vdots \\ e^{jk(x_{n}\sin\theta \cos\varphi +y_{n}\sin\theta \sin\varphi +z_{n}\cos\theta )} \end{array}\right].$$ (23)

To further enhance accuracy, the radiation pattern is synthesized directly from array element patterns to compensate for pattern distortions induced by element mutual coupling and environmental interactions. The governing computational framework can be expressed as:

$$G(\theta ,\phi )\approx E(\theta ,\phi )=[f(\theta ,\phi )][a_{t}]$$ (24)

where [f(θ,ϕ)] is the array unit radiation pattern, which is a 1×N dimensional matrix, $f(\theta ,\varphi )=\left[\begin{array}{ccc} f_{1}(\theta ,\varphi ) & \cdots & f_{n}(\theta ,\varphi ) \end{array}\right]$.

Under the requirement of inter-beam gain equalization, Eq (21) can be further simplified, and the PSO fitness function may be formulated as follows:

$$\text{ Fitness }=\text{ PTE }-\gamma \cdot \text{ Penalty }\;\text{ Penalty }=\sum_{j=1}^{m}{\left|G\left(\theta_{j},\phi_{j}\right)-\frac{1}{m}\sum_{i=1}^{m}G\left(\theta_{i},\phi_{i}\right)\right|}^{2}$$ (25)

where *γ* is the penalty coefficient, which is used to balance PTE and gain balance.

Furthermore, to prevent particle dispersion and escape, a particle expansion boundary is set within the algorithm. When particles collide with this boundary, they cease further outward expansion and remain at it. If the particle expansion boundary is too small, the optimization will fail; if it is too large, the convergence rate will slow down. Appropriate boundary conditions help enhance the algorithm’s convergence speed, enabling particles to find the optimal solution more rapidly. The algorithm exits and terminates after reaching the maximum number of iterations, the global best particle’s weights [*W*] are injected into the synthesis model Eqs (15) and (17), generating excitation vectors that enforce inter-beam gain uniformity.

The algorithm employs a confinement mechanism whereby particles are restricted to a predefined search boundary upon contact. This boundary setting critically determines performance, as excessively tight bounds hinder optimization whereas overly broad ones reduce convergence speed. Appropriate boundary conditions help improve the convergence rate of the algorithm, allowing particles to locate the optimal solution more efficiently. Upon reaching the maximum iteration threshold, the algorithm terminates and outputs the global best particle weight coefficients [*W*] into the excitation calculation models (Eqs 15 and 17), thereby completing the solution for the optimal excitation distribution of the array and achieving a multi-beam balanced-gain radiation pattern.

## Phased array antenna design

In this section, the design, integration, and calibration process of a phased array antenna will be introduced as a validation platform for the algorithm presented in this manuscript.

### Array antenna design

**Fig 4:**
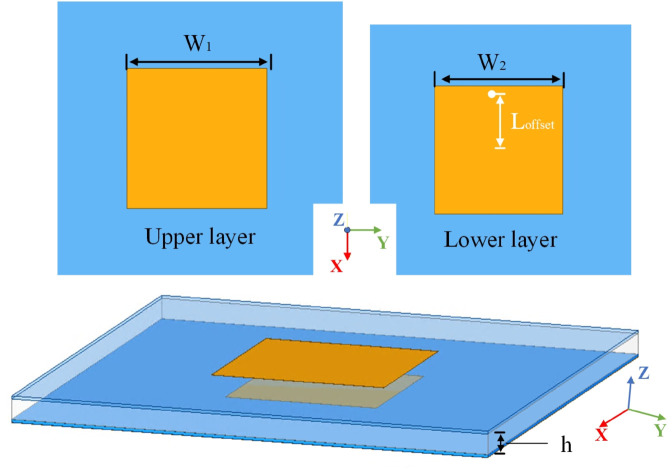
The geometry of the array antenna element.

The array antenna element is based on a two-layer square patch configuration, which serves to enhance the impedance bandwidth while also providing protection for the underlying feeding network. Each dielectric layer has a relative permittivity of 2.6 and a thickness of 0.74 mm. The gap between the two patches is filled with rigid foam with a relative permittivity typically ranging from 1.0 to 1.1, offering structural support. A bottom-mounted coaxial probe feed is adopted for the antenna element. The feed port is connected to the lower patch, which operates as a parasitically coupled element. Energy is transferred via electromagnetic coupling to the upper patch, which acts as the primary radiating element responsible for electromagnetic wave radiation. The configuration of the array antenna unit is as illustrated in Fig 4. The parameter values defined in Fig 4 are as follows (unit: mm) : *W*_1_ = 43.5, *W*_2_ = 38, *L*_*offset*_ = 15.9, *h* = 5.

**Fig 5:**
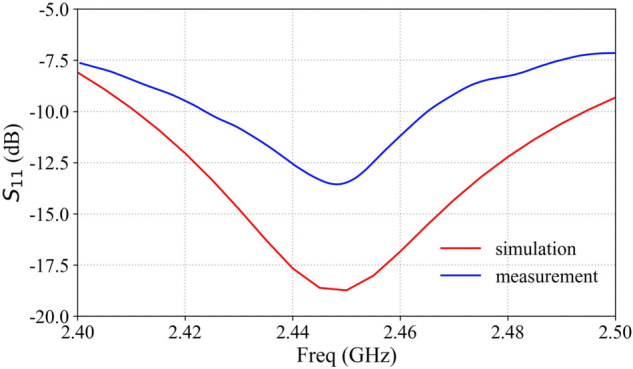
Array antenna element S parameter.

The array antenna element operates primarily at 2.45 GHz. Fig 5 presents the S11 parameters of the antenna unit, demonstrating favorable impedance matching at 2.45 GHz, as observed in the figure.

**Fig 6:**
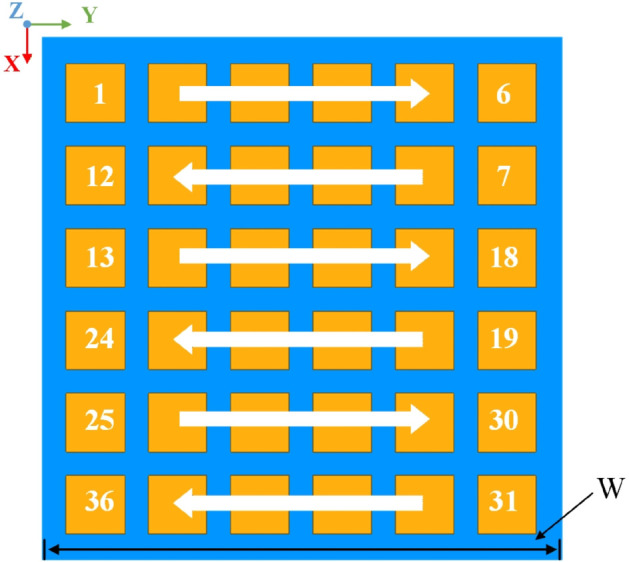
Antenna array geometry layout.

The array antenna elements are arranged in a 6×6 planar configuration on the X-Y plane with a 0.48*λ* spacing (where *λ* is the wavelength at 2.45 GHz), resulting in an overall aperture dimension of 390mm×390mm. The element ports of the array are interconnected through serpentine routing, with the specific element arrangement geometry and port numbering sequence illustrated in Fig 6.

**Fig 7:**
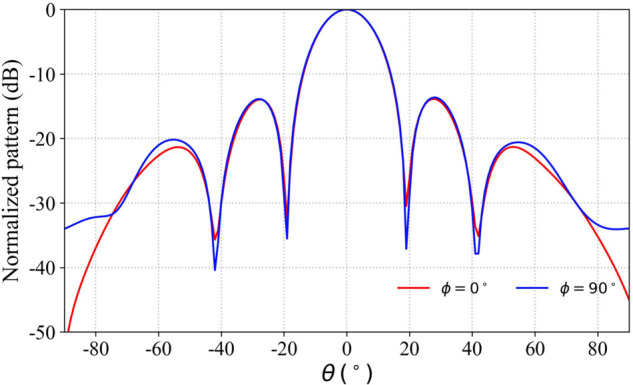
Radiation patterns under uniform amplitude and in-phase excitation.

The normal beam gain of the array antenna is 21 dBi and the beam width is 17 °. The tangential cuts of the radiation patterns in the ‌XOZ‌ and ‌YOZ‌ planes are illustrated in Fig 7.

### Phased array antenna system construction

**Fig 8:**
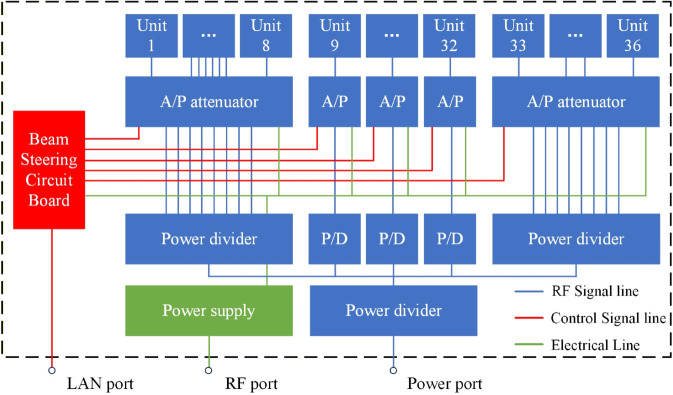
Array system link block diagram.

The phased array antenna system is composed of a beam steering circuit board, RF power divider, A/P attenuator (amplitude phase attenuator), and 12V DC power supply. The integrated link diagram of the array system is shown in Fig 8.

The beam steering circuit board is designed for communication with the host computer and for controlling the A/P attenuators, enabling precise management of each channel in the phased array antenna system. The board is implemented with an Artix-7 FPGA chip, featuring 16 groups of I/O ports that can be scaled to support up to 128 RF channels with millisecond-level response latency. Each I/O port group on the board is connected to the A/P attenuators via ribbon cables. The FPGA translates commands from the host computer into control pulses for the A/P attenuators, generating a synchronous clock signal (40 MHz), an enable signal (TTL logic), and a continuous 16-bit data signal through dedicated pins. This allows each A/P attenuator to simultaneously update the attenuation states of eight RF channels. The beam steering circuit board supports three control modes: individual channel control, grouped control, and centralized control. It is capable of independently adjusting single-channel attenuators, arbitrary groups of attenuators (8 channels per group), and switching between arbitrary beam patterns (with up to 36 channels controlled simultaneously).

**Fig 9:**
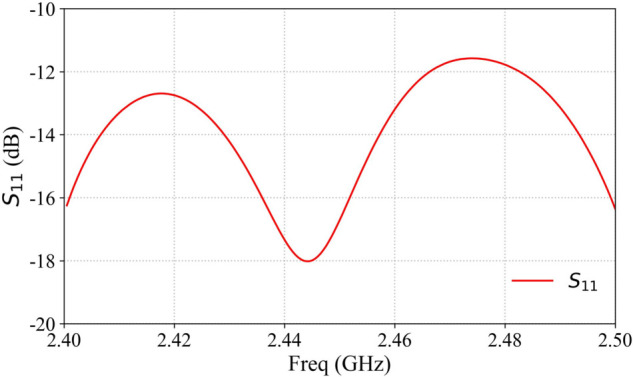
The S parameter of phased array antenna system.

The RF power division network module employs five standardized 1-to-8 power dividers and identical-specification RF cables to uniformly distribute input signals from a single RF channel across 36 output channels connected to A/P attenuator input ports, as illustrated by the blue lines in Fig 8. The primary-stage power divider routes five of its outputs to secondary-stage dividers, while the remaining three outputs are terminated with 50-ohm loads. Each secondary-stage divider directly connects its eight outputs to A/P attenuator input ports, with the fourth secondary divider operating only its first four functional outputs and terminating the latter four with 50-ohm loads. The intrinsic characteristics of the 1-to-8 power dividers introduce 9 dB of attenuation per unit. In this system, the cascaded configuration of primary and secondary power dividers combined with RF cable insertion losses results in an estimated total attenuation of approximately 18 dB for the power division network module. The *S*_11_ measurement results of the phased array antenna are shown in Fig 9.

**Fig 10:**
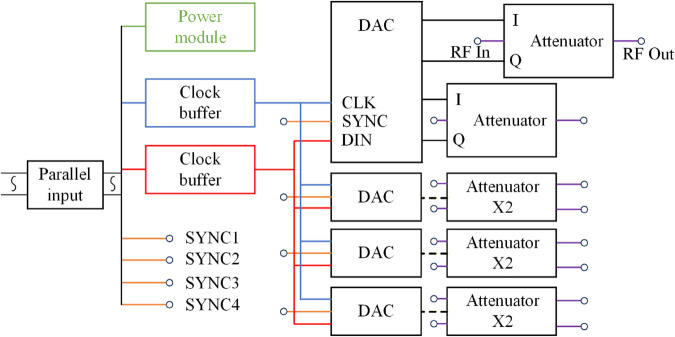
Attenuator composition diagram.

The A/P attenuator is composed of a clock register, a DAC (12 bit digital-to-analog converter), a attenuator IC, a step-down power module, etc. It communicates with the FPGA through a parallel input interface. Its architecture, shown in Fig 10, features eight independently controllable phase-shifting attenuation channels. The attenuator operates over a typical frequency range of 2.2–2.7 GHz, with a peak in-band gain of –11 dB and an attenuation range of 40 dB. Its attenuation characteristics are governed by the voltage levels applied to the I and Q input ports. The relationship between the input port voltages and the phase-shifting attenuation response is expressed as:

$$I(G,\theta )=V_{m}i+1.0V\frac{G}{G_{max}}cos(\theta )\;Q(G,\theta )=V_{m}q+1.0V\frac{G}{G_{max}}sin(\theta )$$ (26)

where $V_{mi}=V_{mq}=1.5V$, *G*_*max*_ = 0.316, $G=10^{\frac{Gain\;Setting(dB)}{20}}$. Calculated according to Eq (26), the input voltage range of the I and Q pins of the phase-shifting attenuator is 0.5–2.5*V*. Through calculation, the voltage control resolution can reach 7e-4 V, the amplitude attenuation resolution is better than 0.1 dB, and the comprehensive phase resolution is better than $1^{\circ }$.

The DAC features 12-bit resolution, 4096-step adjustment granularity, and a pin output voltage range of 0–3V. Independent control of its four voltage output channels is achieved by writing distinct control words and data words to the internal registers. The DAC employs a 16-bit internal register architecture comprising 4 control bits and 12 data bits. A complete register refresh requires 17 clock cycles, with the FPGA providing a 40 MHz clock signal to the DAC. The A/P attenuator integrates four parallel DACs, yielding 16 voltage output pins. Calculated refresh latency for the entire system totals 272 clock cycles (17 cycles/pin × 16 pins), resulting in a microsecond-scale refresh time.

The switching power supply steps down the input voltage to 12 V to power the phased array antenna system. Within the amplitude-phase attenuator circuitry, the voltage is further regulated to 9 V, 5 V, and 3.3 V for powering onboard electronic components. Critically, to ensure accurate digital signal level recognition by the ICs in the amplitude-phase attenuator, the signal ground (SGND) and power ground (PGND) must be interconnected in series.

**Fig 11:**
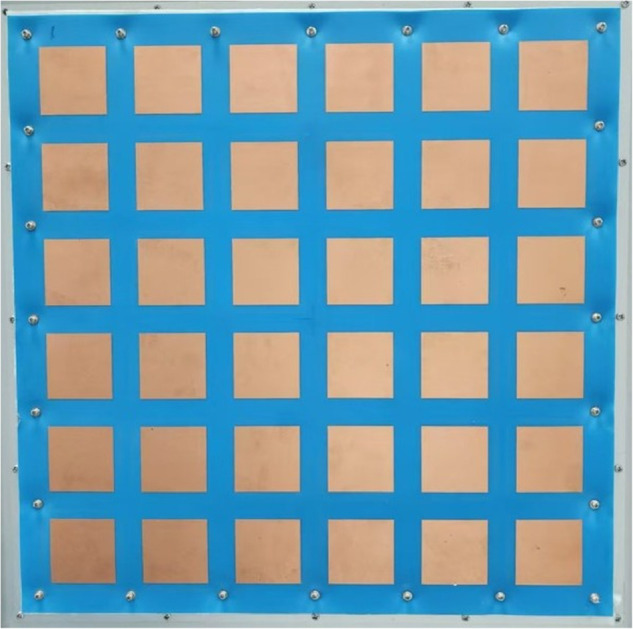
Photograph of the phased array antenna system (Front Side).

**Fig 12:**
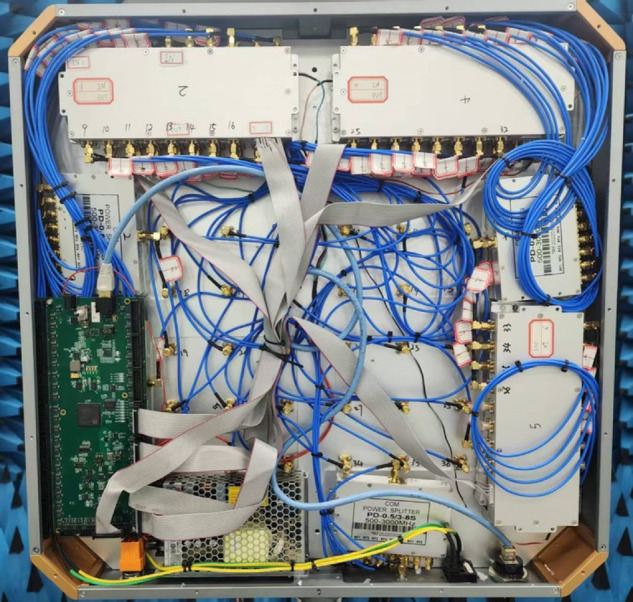
Photograph of the phased array antenna system (Back Side).

The components of the phased array antenna system are integrated as shown in the Figs 11 and 12.

### Calibration of phased array antenna system

**Fig 13:**
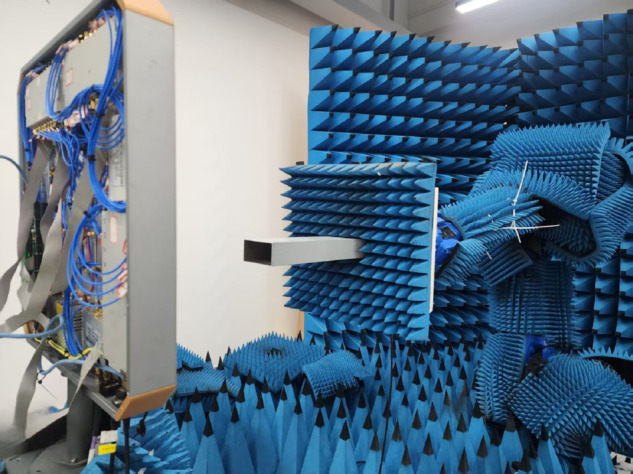
Phased array antenna system calibration setup.

The calibration of a phased array antenna system is used to compensate for amplitude and phase inconsistency errors in each RF channel caused by factors such as device variations, cable differences, array coupling, and operating environments. Calibration corrects these errors to ensure all antenna elements achieve a uniform amplitude and phase reference. Typically, the calibration of a phased array antenna system is performed in two steps [38]. The first step involves the consistency calibration of the radiation reference of the antenna array, where the radiation reference refers to the amplitude-phase state that enables the array antenna to generate the normal beam. The second step focuses on the calibration of attenuation characteristics of the array antenna attenuators. A typical near-field calibration scenario of a phased array antenna system is shown in Fig 13.‌‌‌‌‌‌‌‌

**Fig 14:**
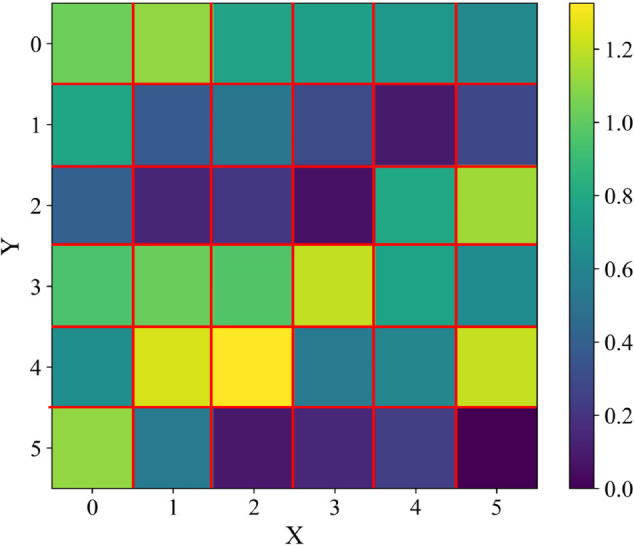
Reference Distribution Map of Channel Consistency.

A waveguide probe operating at 2.45 GHz is positioned on a plane located 3*λ* away from the radiating elements of the array antenna. Each individual antenna element is activated sequentially using an identical reference signal, while the waveguide probe measures the radiated electric field value in front of the active element.‌‌ By performing channel consistency calibration across all 36 elements, the response deviation of each antenna element under typical uniform amplitude and phase excitation can be quantified, as shown in Fig 14. The 36 grids in the figure represent the amplitude distribution across the 36 RF channels on the reference plane under equal-amplitude and in-phase excitation, with the lowest amplitude among the sampled points taken as the relative reference.

**Fig 15:**
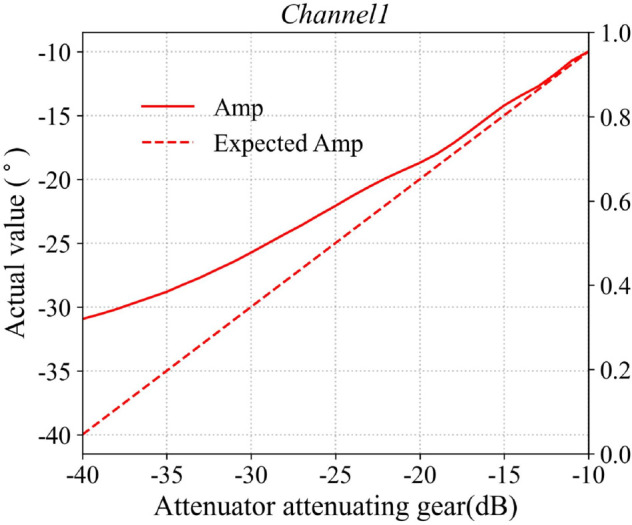
Attenuator phase response performance curve.

**Fig 16:**
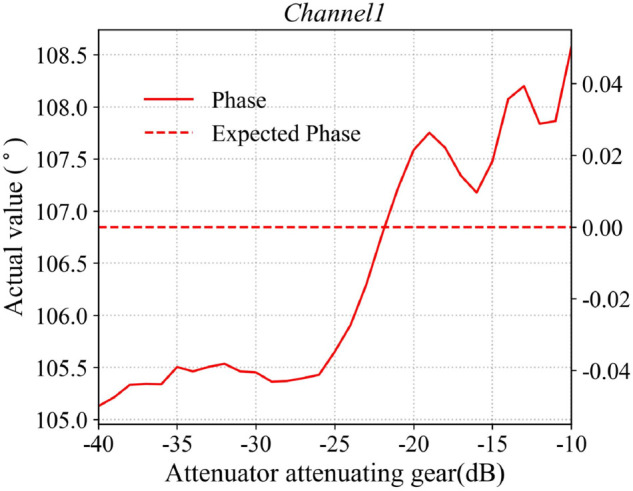
Attenuator phase response performance curve.

Typically, the attenuation response of amplitude-phase attenuators exhibits nonlinear behavior, necessitating the calibration of its attenuation-phase-shift characteristics under typical operating conditions. The host computer sends batch-control commands to the phased array antenna system, sequentially attenuating the RF signals with equal step sizes, while a waveguide probe simultaneously records the response characteristics of the antenna element’s radiated field throughout the entire attenuation process. In this paper, the response curve of the first channel is selected as representative data, with the results shown in Figs 15 and 16. The figures compare the measured response of the attenuator with its ideal response. The X-axis represents the attenuation value commanded by the host computer, while the Y-axis indicates the actual response of the attenuator. As illustrated, the effective attenuation range of the A/P attenuator is approximately 31 dB. By constructing a 36-channel attenuator calibration database and applying a look-up-table (LUT) approach, a precise mapping between the command values and the actual responses of the A/P attenuator is established. This enables device calibration by compensating for the inherent nonlinearity and inter-channel variations of the control system.

**Fig 17:**
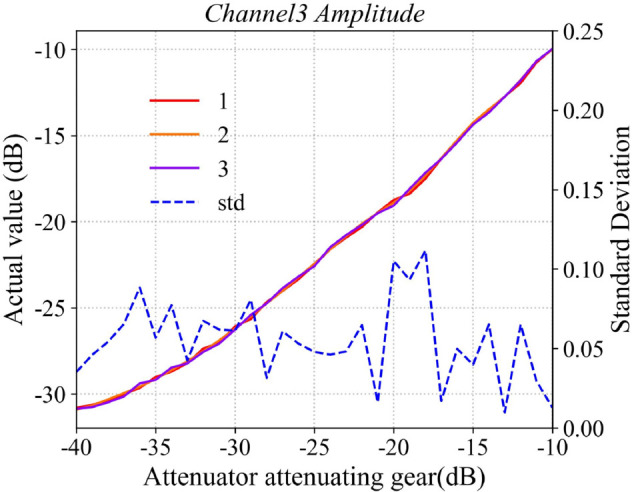
Comparison of repeatability results of the amplitude response of attenuators.

**Fig 18:**
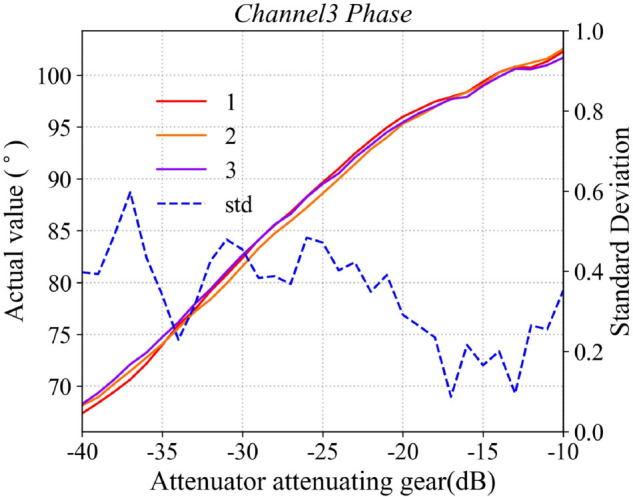
Comparison of repeatability results of the phase response of attenuators.

To evaluate the repeatability of the calibration procedure, the phased array antenna system underwent three cycles of disassembly, reassembly, power-up, and recalibration. During each calibration, the relative position between the receiving antenna and the transmitting array surface remained consistent. The amplitude phase (A/P) attenuators were controlled to vary only in amplitude while maintaining the output phase at $0^{\circ }$. The measured amplitude phase response curves for all 36 channels were recorded. The three amplitude phase response curves for the third RF channel are presented in the paper as shown in Figs 17 and 18. The results indicate good consistency of the A/P attenuator performance: the standard deviation of the amplitude across the three measurements is less than 0.15 dB, and that of the phase is below 0.5∘. These findings demonstrate that the calibration procedure is repeatable and that the A/P attenuators exhibit satisfactory stability.

The calibrated channel consistency parameters and the attenuator response correction tables were imported into the array antenna control software. The far field radiation pattern of the array antenna was then measured in a semi anechoic chamber using a robotic planar near field scanning system. Prior to testing, the robotic system was fully wrapped with absorbing material to avoid metallic reflections in front of the antenna under test. Environmental interference, which typically has a pronounced effect on weak signals, can manifest in the measured results as reduced null depth and increased sidelobe fluctuation. During testing, a suitable RF link was configured and the intermediate frequency (IF) bandwidth of the vector network analyzer was appropriately set to ensure a high dynamic range for the measurement system. The sampling interval of the receiving probe was set to 0.48λ, the probe speed was set to 30 mm/s, and the scan plane measured 1.2 m × 1.2 m (approximately 10λ×10λ) at a distance of 3λ from the antenna aperture.

**Fig 19:**
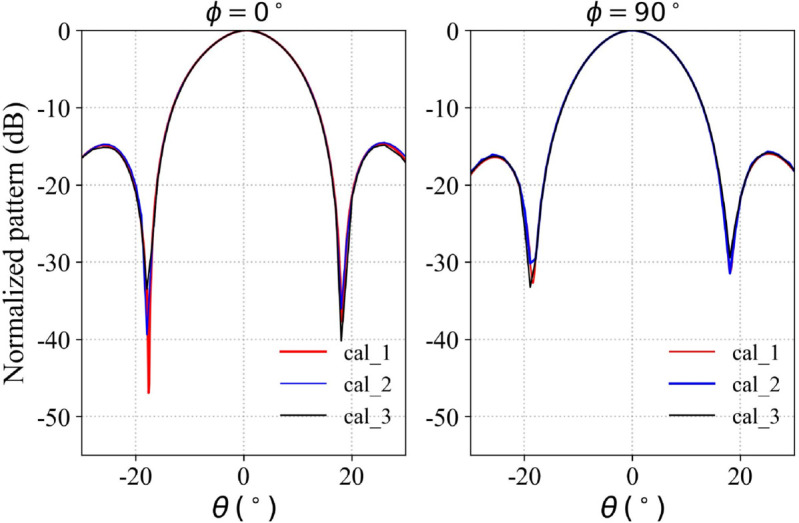
Comparison of repeatability results of the phase response of antenna radiation pattern.

Keeping the test setup and conditions unchanged, the phased array antenna was powered off and then restarted. The beam pattern corresponding to the same set of array excitations was measured three times, and the results were recorded. A comparison of the three measurements is shown in Fig 19. The results indicate that the phased array antenna operates with good stability: across the repeated measurements, the main lobe and the half power beamwidth remained consistent, while only minor fluctuations were observed in the first null depth and the first sidelobe region. These findings demonstrate that both the phased array antenna and the measurement system exhibit a high degree of stability.

**Fig 20:**
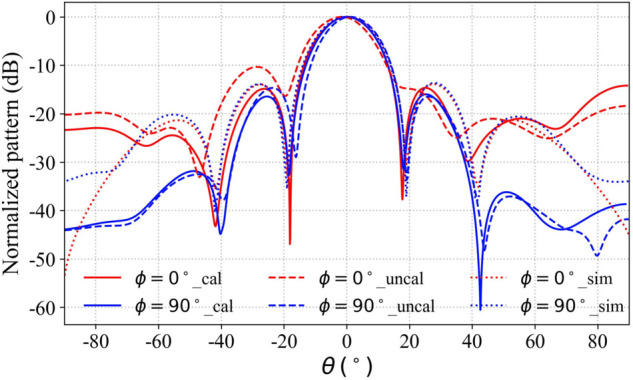
Measured radiation patterns of the calibrated array antenna.

The comparison between the measured and simulated radiation patterns of the array antenna before and after calibration is shown in the corresponding figure. The experimental results presented in Fig 20 indicate that the calibrated antenna radiation pattern shows good agreement with the simulation. These results demonstrate that the calibration framework described above successfully mitigates amplitude-phase errors in the RF chain of the phased array system. At 2.45 GHz, the simulated gain is 21 dBi, while the measured gain is 20.6 dBi, with a half-power beamwidth of $16^{\circ }$. In contrast, the radiation pattern prior to calibration deviates significantly from the simulation.

It should be noted that the calibration of the amplitude-phase response of the RF attenuator in this work was performed using a 1 dB attenuation step, and the limited resolution of the attenuator’s response curve can introduce some error into the final radiation pattern. Furthermore, the finite size of the scan plane in the near-field measurement system imposes constraints on accuracy. When the main beam of the antenna is not directed toward the center of the scan plane, the measurement system captures less energy from the main lobe, which leads to reduced precision in the measured results.

## Analysis of the proposed method

**Fig 21:**
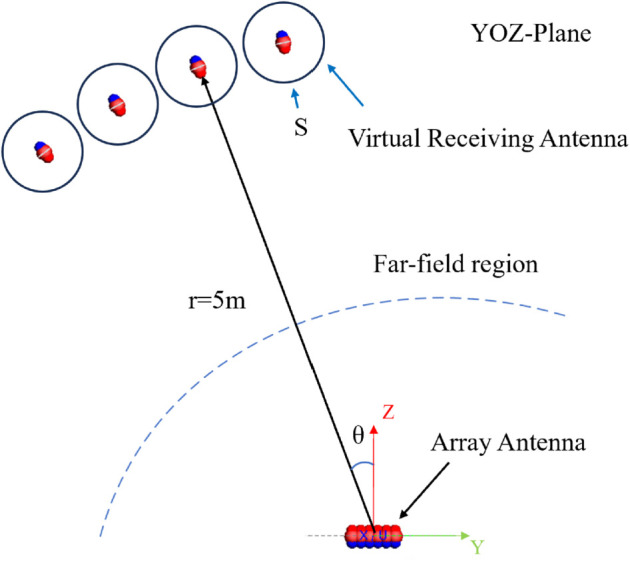
Simulation model layout illustration.

The results of all the examples in the paper are calculated on a 64-bit workstation (Intel Xeon Gold-6148@2.40 GHz), and the code runs on the Python 3.9 platform. The advantage of the proposed algorithm is that it uses equation calculation instead of the simulation model to build the scattering matrix of transmitting and receiving antennas. Using the array mentioned above, four receiving antennas are deployed in the far-field region at a distance of 5 m from the array antenna, forming a 4-group 36  +  1-ports power transfer model, where the receiving antennas have the same structure as the array antenna element. The azimuthal positions of the receiving antennas are set at ($\theta =10^{\circ },\phi =270^{\circ }$), ($\theta =20^{\circ },\phi =270^{\circ }$), ($\theta =30^{\circ },\phi =270^{\circ }$), and ($\theta =40^{\circ },\phi =270^{\circ }$), respectively. In Fig 21, the position relationship between the array antenna and the receiving antenna is shown. Fig 21 illustrates the positional relationship between the array antenna and the receiving antennas. Prior to the proposed approach, determining the array excitations for multi-beam patterns via the MMPTE method required constructing the simulation model depicted in the figure and obtaining the S matrix of the multi antenna system using simulation software. In contrast, the present method replaces the physical model of the receiving antennas with a spherical electric field **E** defined over a closed surface that encloses them. The positional relationship of the four receiving antennas relative to the array antenna can then be characterized through spatial coordinate transformations of this spherical field distribution.

**Fig 22:**
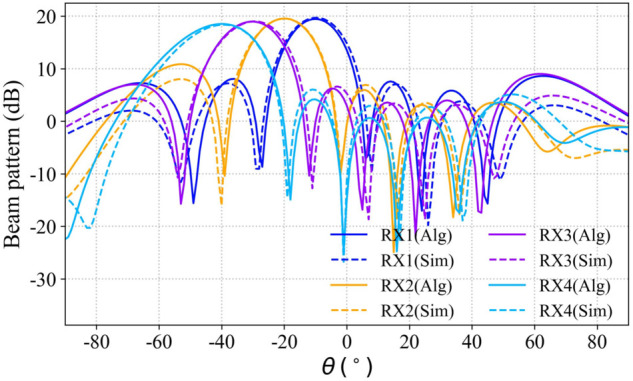
Comparison of the pattern constructed by algorithm and simulation model.

The S parameter matrices of the multi antenna system were extracted using both the simulation software and the proposed method, respectively, and the array excitations were then calculated using Eq (17). In Fig 22, the solid lines represent the radiation patterns constructed from the S parameters obtained via the proposed method (Alg), while the dashed lines correspond to those derived from the S parameters extracted via the simulation model (Sim). The virtual receiving antenna numbers in the legend match the annotations in Fig 21, indicating the beam patterns constructed based on the virtual receiving antenna at the corresponding position. The figure shows that the beams synthesized using the proposed method align well with those generated by the simulation model in the main lobe region, demonstrating that the proposed method can effectively substitute for the simulation based solution process.

**Fig 23:**
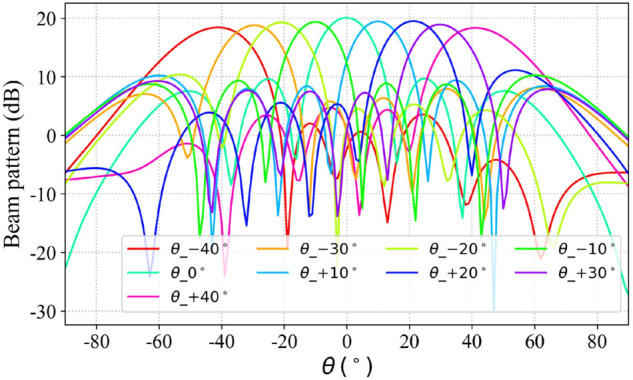
Simulation scanning beam radiation pattern of array antenna (XOZ-plane).

**Fig 24:**
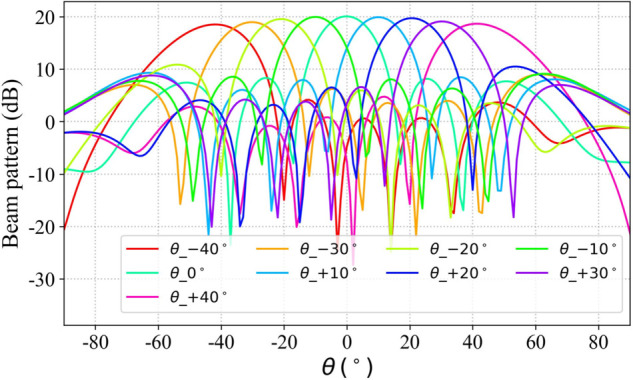
Simulation scanning beam radiation pattern of array antenna (YOZ-plane).

The proposed algorithm was employed to synthesize scanning beams at $\pm 40^{\circ }$ using the phased array antenna model. The resulting scanning beams along with their simulated counterparts in the XOZ and YOZ planes are illustrated in Figs 23 and 24, respectively. This method is capable of synthesizing array scanning beams directed toward arbitrary positions. Simulation results indicate that the beam steering accuracy achieved by this approach can reach 0.1° or better.

According to uniform linear array theory, the $\theta_{grating}$ at which a grating lobe occurs is determined by the formula $\sin\theta_{grating}=\sin\theta_{0}$  +  mλ/d, where $\theta_{0}$ is the main-lobe steering angle and m is a nonzero integer. The two-dimensional array antenna discussed in this paper is arranged with half-wavelength equidistant. At the center frequency of 2.45 GHz, the element spacing is set to 0.48*λ*. Even when steering to the maximum target angle ($\pm 40^{\circ }$), calculation shows that for the first-order grating lobe (m = –1), the value of $\sin\theta_{grating}$ is less than –1, which lies outside the visible space. Therefore, no grating lobe appears within the entire steering range of the array.

**Fig 25:**
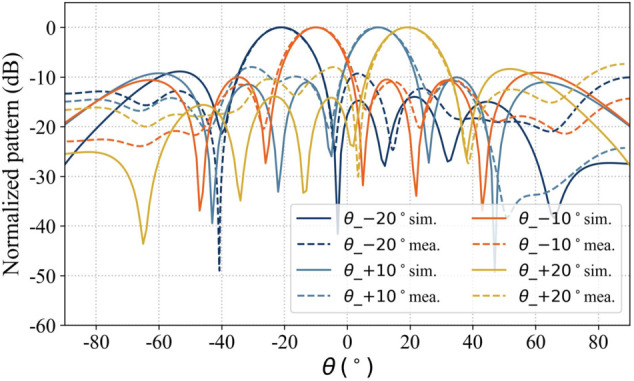
Measured scanning beam radiation pattern of array antenna (XOZ-plane).

**Fig 26:**
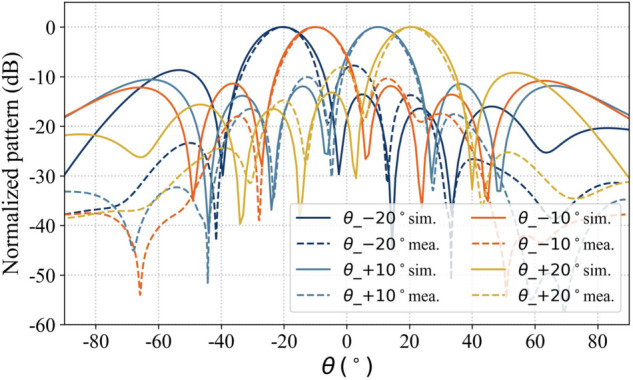
Measured scanning beam radiation pattern of array antenna (YOZ-plane).

Four sets of simulated radiation patterns were selected from the XOZ and YOZ planes in Figs 23 and 24, and the corresponding measured radiation patterns were obtained using a robotic near-field antenna measurement system [39], as shown in Figs 25 and 26. The results indicate that the measured main lobes of the beams show good agreement with their simulated counterparts.

Two multi-beam synthesis case studies based on the proposed method in this paper are presented below to demonstrate the method’s effectiveness. Case 1, consider synthesizing four beams with identical gains, directed toward the azimuthal angles ($\theta =20^{\circ },\phi =270^{\circ }$), ($\theta \;=\;20^{\circ },\phi =90^{\circ }$), ($\theta =50^{\circ },\phi =270^{\circ }$), and ($\theta =50^{\circ },\phi =90^{\circ }$) in the YOZ plane. Four virtual receiving antennas are positioned at these specified angles, and the four-beam radiation patterns are synthesized using Eqs (14) and (17). Since the algorithm prioritizes maximizing transmission efficiency when computing the array excitations, this results in an imbalance in the gain distribution among the four beams. To address this issue, the method proposed in Section is applied to adjust the gain distribution weighting coefficient matrix in Eq (15).

**Fig 27:**
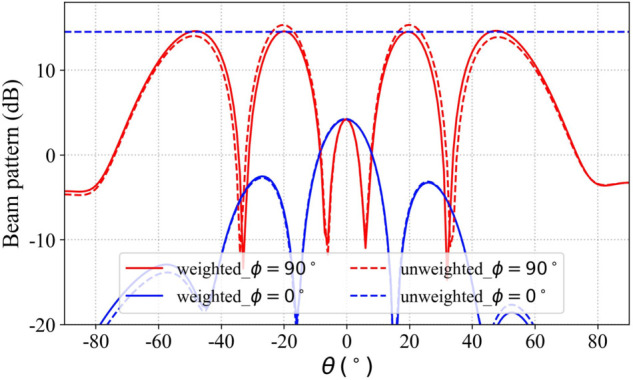
Comparison of the pattern before and after gain equalization optimization.

Table 1 shows the array excitation distributions of case 1 before and after weighting, while Fig 27 provides a comparison of the radiation patterns before and after weighting for case 1. The experimental results demonstrate that following optimization of the gain allocation weighting matrix, the array maintains beam gains at a consistent level of approximately 14.63 dBi, with inter-beam gain variation measured below 0.05 dB, achieving exceptional gain uniformity across all synthesized radiation patterns as theoretically predicted.

**Table 1:** Distributions of excitations.

Port	Case 1		Case 2	
	Weighted	Unweighted	Weighted	Unweighted
1	0.27∠−6	0.27∠−5	0.28∠−2	0.28∠−2
2	0.14∠169	0.11∠170	0.16∠−4	0.16∠−4
3	0.10∠173	0.13∠174	0.24∠−2	0.23∠−2
4	0.09∠173	0.12∠171	0.21∠−5	0.21∠−5
5	0.15∠176	0.12∠178	0.18∠0	0.18∠1
6	0.28∠−2	0.29∠−2	0.28∠−2	0.28∠−1
7	0.27∠0	0.27∠28	0.04∠72	0.04∠74
8	0.16∠−150	0.14∠−150	0.04∠158	0.04∠155
9	0.04∠168	0.06∠177	0.02∠96	0.03∠95
10	0.04∠171	0.06∠−172	0.03∠93	0.03∠92
11	0.16∠−151	0.14∠−151	0.05∠161	0.05∠158
12	0.26∠19	0.26∠19	0.05∠−165	0.05∠61
13	0.18∠19	0.19∠19	0.23∠−165	0.23∠−165
14	0.09∠−149	0.07∠−145	0.09∠−179	0.09∠−179
15	0.08∠162	0.10∠168	0.17∠−169	0.17∠−169
16	0.08∠173	0.10∠174	0.15∠−174	0.15∠−174
17	0.08∠−153	0.07∠−148	0.11∠−174	0.11∠−174
18	0.18∠16	0.18∠15	0.22∠−167	0.22∠−168
19	0.18∠15	0.18∠14	0.22∠−170	0.22∠−169
20	0.07∠−157	0.05∠−154	0.10∠−171	0.10∠−170
21	0.09∠175	0.11∠176	0.17∠−175	0.17∠−175
22	0.08∠163	0.10∠168	0.16∠−171	0.16∠−170
23	0.08∠−151	0.06∠−146	0.10∠180	0.10∠−180
24	0.18∠16	0.18∠16	0.22∠−166	0.22∠−165
25	0.25∠24	0.25∠25	0.04∠62	0.04∠59
26	0.16∠−152	0.14∠−151	0.04∠165	0.04∠169
27	0.04∠167	0.06∠−176	0.03∠77	0.03∠75
28	0.04∠175	0.06∠−176	0.03∠105	0.02∠108
29	0.16∠−149	0.13∠−149	0.04∠156	0.04∠160
30	0.25∠27	0.25∠27	0.04∠77	0.03∠75
31	0.30∠0	0.31∠0	0.30∠0	0.30∠0
32	0.15∠177	0.13∠178	0.17∠2	0.17∠2
33	0.09∠173	0.12∠171	0.23∠−4	0.23∠−4
34	0.09∠173	0.12∠174	0.22∠−2	0.22∠−2
35	0.14∠171	0.12∠173	0.17∠−3	0.17∠−3
36	0.29∠−4	0.29∠−3	0.29∠0	0.29∠0

**Fig 28:**
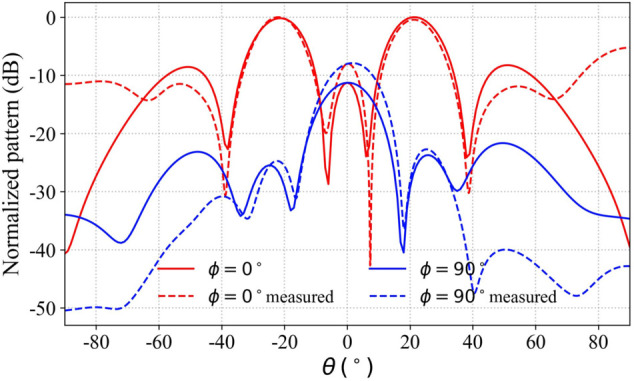
Comparison of the simulation and measured radiation pattern in case 2.

Case 2 synthesizes two beams with identical gains, directed toward the azimuthal angles ($\theta =20^{\circ },\phi =0^{\circ }$), ($\theta =20^{\circ },\phi =180^{\circ }$) in the XOZ plane. The beam gains of the array are maintained at a consistent level of approximately 17 dBi with a gain variation of less than 0.1 dB between the beams. The array excitation distributions of case 2 before and after weighting are shown in Table 1. The measurement results of the weighted excitation main lobe beam are consistent with the simulation results, as shown in Fig 28. The weighted value distribution is shown in Table 2, where $\left[W]\;=\;diag(w_{1},w_{2},\cdots ,w_{m}\right)$.

**Table 2:** Weighted value distribution.

	$w_{1}$	$w_{2}$	$w_{3}$	$w_{4}$
Case 1	2.1712	2.1767	2.9257	3
Case 2	1.9905	2		

**Fig 29:**
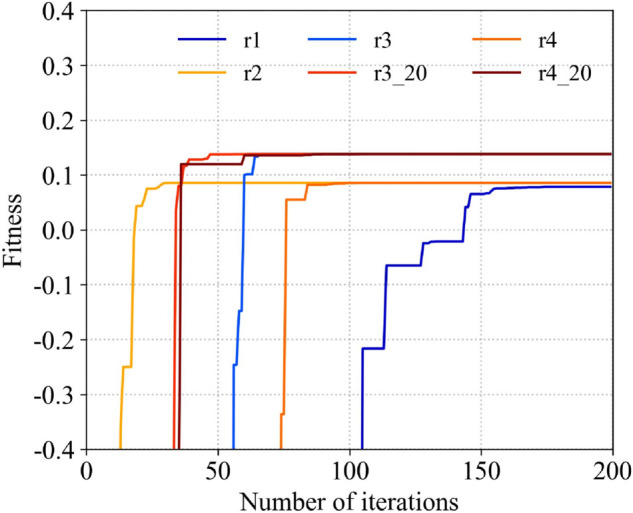
The relationship between PSO initialization conditions and convergence.

Taking the process of calculating the weighted value of case 1 as an example, the applicability of different inertia factors and learning factors with the number of iteration rounds under the condition of initializing 40 particles is plotted in the figure, as shown in Fig 29. The definition of the legend in the figure is given in Table 3. It can be seen from the figure that the selection of the inertia factor and the learning factor will affect the speed of the convergence of applicability. Setting the inertia factor as [0.5-2.5] and the learning factor as [0.5-2.5] is a relatively balanced choice.

**Table 3:** Parameter definitions in Fig 29.

	$w_{min}$	$w_{max}$	$c_{1,min}$	$c_{1,max}$	$c_{2,min}$	$c_{2,max}$	reset rate
r1	0.4	0.9	2.0	2.0	2.0	2.0	10%
r2	0.5	0.5	0.5	2.5	0.5	2.5	10%
r3	0.5	0.5	2.0	2.0	2.0	2.0	10%
r3_20	0.5	0.5	2.0	2.0	2.0	2.0	20%
r4	0.4	0.9	0.5	2.5	0.5	2.5	10%
r4_20	0.4	0.9	0.5	2.5	0.5	2.5	20%

**Fig 30:**
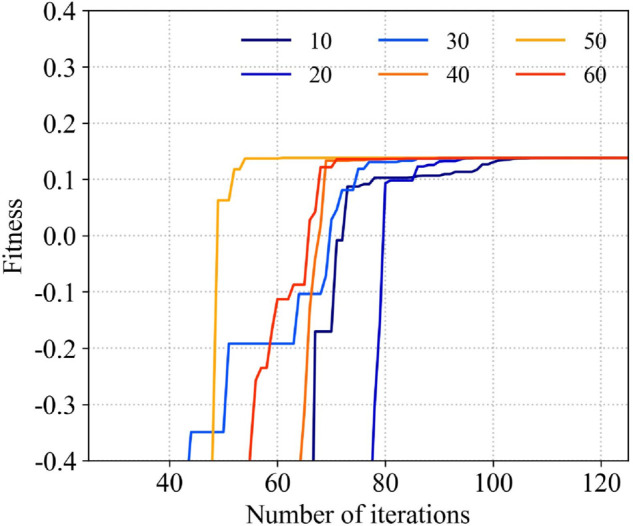
The relationship between the number of particles and the convergence rate.

**Fig 31:**
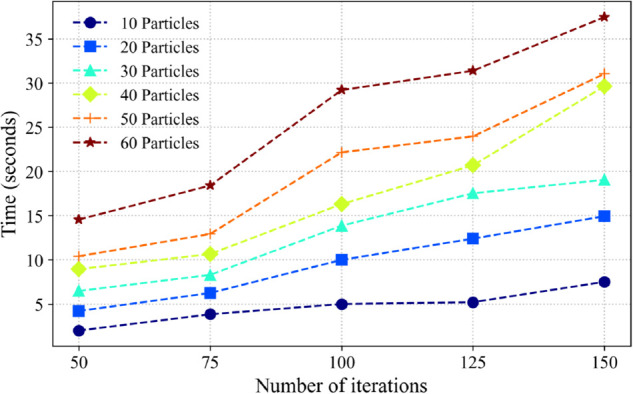
Algorithm running time curve.

The applicability of different particle quantities with the variation of iteration rounds is plotted in the graph, as shown in Fig 30. It can be seen that under the same parameters, the relationship between the number of initialized particles and the number of iterations required for the applicability to tend to be stable. In the application of the algorithm, it is necessary to set the number of initialization particles and the number of iterations reasonably. Combine and match different particle quantities and iteration rounds, and plot the time taken by the algorithm in Fig 31. Taking into account the convergence time of the algorithm and the size of resource consumption comprehensively, the parameters used by PSO in this example are shown in Table 4 as follows. The running time of the algorithm is about 7.8 s.

**Table 4:** Parameter using in case 1.

$w_{min}$	$w_{max}$	$c_{1,min}$	$c_{1,max}$	$c_{2,min}$	$c_{2,max}$
0.4	0.9	0.5	2.5	0.5	2.5
particles	bounds	max iterations	diversity threshold	reset rate	*γ*
50	[0,3]	75	1e-3	20%	1

Compared to the conjugate beamforming method, the LCMV beamforming method, and the gradient descent method, our approach reduces the runtime by 69%, 68%, and 85%, respectively. The performance comparison is shown in Table 5. While the memory efficiency remains on the same order of magnitude, the beam gain variation metric is significantly better than that of the other methods. Although the convex optimization approach achieves faster computation, its beam gain variation performance is slightly inferior to our algorithm. When compared with results extracted from published literature under similar performance metrics, the proposed method remains competitive across several key indicators, while avoiding the substantial offline training cost required by deep learning-based methods.

**Table 5:** Algorithm performance comparison.

Methoed	Array scale	Running time	Memory efficiency	Beam difference
Proposed	6×6	7.8 s	47.22 MB	±0.1 dB
Conjugate beamforming	6×6	24.8 s	51.10 MB	±1.5 dB
LCMV	6×6	23.4 s	30.42 MB	±2 dB
Convex optimization	6×6	1.4 s	5.44 MB	±1 dB
Gradient descent	6×6	51.5 s	61.50 MB	±3 dB
CPDD [40]	1×20	1314 s	-	±0.1 dB
Deep learning [41]	16×16(Large array)	140 s	-	±5 dB

**Fig 32:**
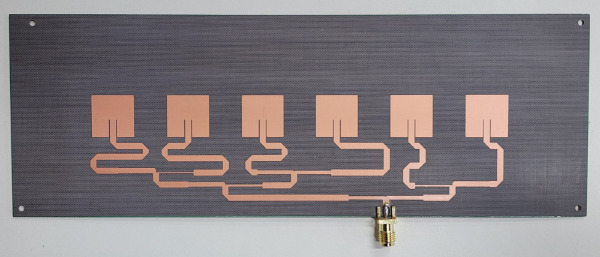
Photograph of the six-element array antenna.

To further demonstrate the effectiveness of the proposed method, a six element linear patch array antenna capable of generating four beams is designed, as illustrated in Fig 32.

The objective of this antenna design is to produce a directional four beam radiation pattern. The overall dimensions of the antenna are 70 mm×200 mm, with each element measuring 14.32 mm × 14.32 mm. The dielectric substrate has a relative permittivity of 3.0 and a thickness of 0.76 mm, and the operating frequency is 5.8 GHz. Using the method proposed in this work, the excitation distribution of the array elements is calculated, as summarized in Table 6.

**Table 6:** Excitation distribution of the six-element array antenna.

Port	Array excitation
1	0.56∠173
2	0.26∠–175
3	0.30∠–1
4	0.31∠179
5	0.23∠6
6	0.62∠0

**Fig 33:**
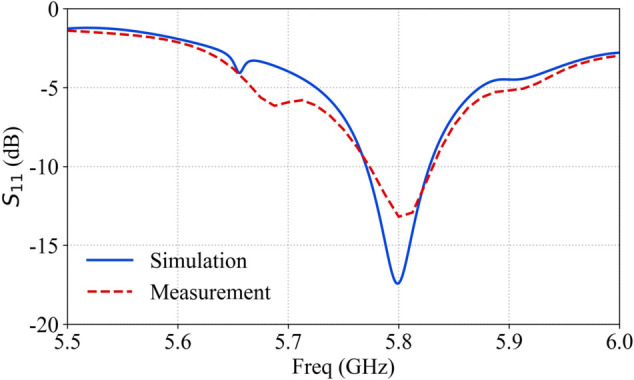
The S parameter of the six-element array antenna.

Based on the calculated excitation values in Table 6, a suitable feeding network is designed using a series feeding configuration. The measured *S*_11_ parameter of the antenna is presented in Fig 33.

**Fig 34:**
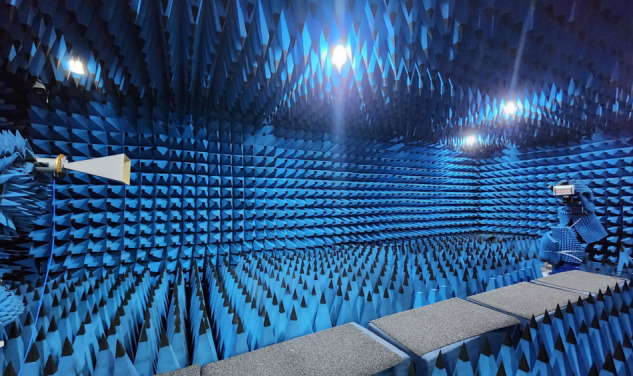
Test scenario for far field measurement.

A dual robot far field antenna measurement system was employed, with a horn antenna mounted at the transmitting end and the antenna under test (AUT) placed at the receiving end. The *S*_21_ parameter was analyzed using a vector network analyzer to obtain the far field radiation pattern of the antenna, and the gain of the AUT was determined using the gain comparison method. The test setup is shown in Fig 34 below.

**Fig 35:**
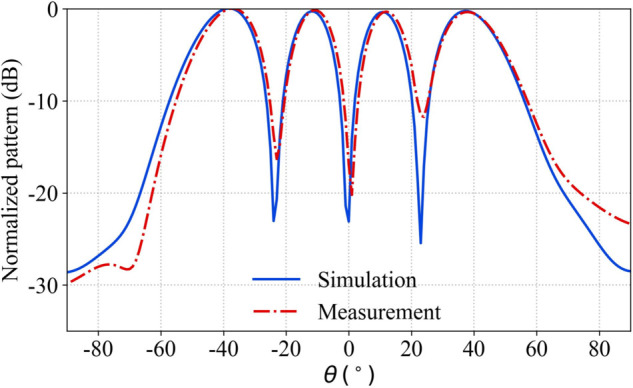
The radiation pattern of the six-element array antenna.

A comparison between the measured and simulated four beam radiation patterns is presented in Fig 35. The four beams achieve a maximum gain of 9.0 dBi, with a measured gain variation among the beams of about 0.5 dB. The beams are directed toward *θ* = –39°, –11°, 11°, and 39°, respectively. The measurement results of the patch array antenna demonstrate that the proposed method effectively accomplishes the synthesis of multi beam radiation patterns, and the workflow and outcomes meet the expected requirements.

The method relies fundamentally on the radiation pattern of a reference element. At higher frequencies (e.g., millimeter-wave or terahertz bands), although shorter wavelengths introduce physical size challenges, the method remains applicable in principle. It may even exhibit greater advantages in certain performance aspects due to the increased electrical size. In summary, the proposed method possesses the inherent capability for scaling to larger arrays and higher frequencies, but its practical application necessitates integration with advanced array architectures, manufacturing processes, and intelligent algorithms.

## Conclusion

This paper presents a comprehensive methodology for multi-beam pattern synthesis in array antennas through the integration of the Lorentz reciprocity theorem, wireless power transmission theory, and particle swarm optimization algorithms, with complete mathematical derivation of governing equations. Further, to validate the methodology, a 2.45 GHz phased array antenna featuring a dual-layer feed unit architecture has been designed and fabricated, with detailed exposition of its internal configuration, operational principles, and radio-frequency channel calibration procedures. The proposed algorithm has been experimentally validated through successful synthesis and measurement of spatial 2D scanning beams, as well as balanced-gain dual-beam and quad-beam radiation patterns. A six-element patch antenna array was also designed to demonstrate the performance of the method in a fabricated antenna prototype. Furthermore, the influence of some parameters in the particle swarm optimization algorithm on the performance and efficiency of the algorithm is discussed in the manuscript, and presents the parameters used in the method proposed in this paper. Experimental results show that the method can accurately form beams with a gain variation of less than 0.1 dB among beams. This pattern synthesis methodology, requiring only array element radiation patterns and the position of the beam target as input, exhibits universal applicability for scan beam synthesis, multi-beam generation with gain equalization, establishing an effective beamforming framework for practical phased array system implementations.
